# Ultrafast control of fractional orbital angular momentum of microlaser emissions

**DOI:** 10.1038/s41377-020-00415-3

**Published:** 2020-10-21

**Authors:** Zhifeng Zhang, Haoqi Zhao, Danilo Gomes Pires, Xingdu Qiao, Zihe Gao, Josep M. Jornet, Stefano Longhi, Natalia M. Litchinitser, Liang Feng

**Affiliations:** 1 Department of Electrical and Systems Engineering, University of Pennsylvania, Philadelphia, PA 19104 USA; 2 Department of Electrical and Computer Engineering, Duke University, Durham, NC 27708 USA; 3 Department of Materials Science and Engineering, University of Pennsylvania, Philadelphia, PA 19104 USA; 4 Department of Electrical and Computer Engineering, Northeastern University, Boston, MA 02115 USA; 5 Dipartimento di Fisica, Politecnico di Milano and Istituto di Fotonica e Nanotecnologie del Consiglio Nazionale delle Ricerche, Piazza L. da Vinci 32, I-20133 Milano, Italy; 6 Instituto de Fisica Interdisciplinar y Sistemas Complejos IFISC (CSIC-UIB), Palma de Mallorca, Spain

**Keywords:** Microresonators, Semiconductor lasers

## Abstract

On-chip integrated laser sources of structured light carrying fractional orbital angular momentum (FOAM) are highly desirable for the forefront development of optical communication and quantum information–processing technologies. While integrated vortex beam generators have been previously demonstrated in different optical settings, ultrafast control and sweep of FOAM light with low-power control, suitable for high-speed optical communication and computing, remains challenging. Here we demonstrate fast control of the FOAM from a vortex semiconductor microlaser based on fast transient mixing of integer laser vorticities induced by a control pulse. A continuous FOAM sweep between charge 0 and charge +2 is demonstrated in a 100 ps time window, with the ultimate speed limit being established by the carrier recombination time in the gain medium. Our results provide a new route to generating vortex microlasers carrying FOAM that are switchable at GHz frequencies by an ultrafast control pulse.

## Structured light: Fractional on-chip source

An integrated vortex laser that is capable of emitting 1495 nm light with tunable fractional orbital angular momentum (OAM) could prove useful for next-generation optical communication systems. Zhifeng Zhang and co-workers from the US, Italy, and Spain fabricated an InGaAsP microring laser embedded in Si_3_N_4_ using electron-beam lithography, inductively coupled plasma etching, and plasma-enhanced chemical vapour deposition. By spatially selective optical pumping with 140-fs duration, 800-nm wavelength laser pulses, an InGaAsP waveguide on the chip couples with the microring at three points and feeds it with dynamically varying optical gain to tune the state of OAM of the microring laser’s output between a charge value of 0 and +2 within 100 ps. The tuning speed and range of achievable OAM values could likely be improved in the future, according to the researchers.

## Introduction

The vectorial nature of light empowers full control of topological features with spatially phase-variant fields, revealing vortex beams carrying orbital angular momentum (OAM) and spin angular momentum (SAM) in addition to the well-known linear momentum^1^. Owing to the intriguing properties of optical vortices, OAM beams have found numerous applications in particle guiding and trapping, optical communication, quantum computation, holography and high-precision imaging, and novel light–matter interactions with topological materials^2–8^. Only quite recently have optical beams carrying a *fractional* orbital angular momentum (FOAM)^9,10^ attracted considerable attention for their unusual properties and potential applications^11,12^. FOAM beams display subtle topological features^13^, and their propagation manifests some unusual mathematics of transfinite numbers^14^.

The total angular momentum associated with electromagnetic (EM) fields in a homogeneous medium, such as free space, is

1 $$\overrightarrow J = \mathop {\int}\nolimits {\rm{d}}\overrightarrow r \varepsilon _0\overrightarrow r \times( {\overrightarrow E \times \overrightarrow B })$$

where *r* is the position, *ε*_0_ is the dielectric permittivity of free space, and $\overrightarrow E$ and $\overrightarrow B$ are the electric field and magnetic flux density of the EM wave, respectively. In the paraxial limit, the phase variation and polarization state of an optical beam are uncoupled, featuring two independent types of angular momenta: OAM (*L*) and SAM (*S*):

2 $$\begin{array} {ccc}\overrightarrow J \,=\, \varepsilon _0\mathop {\sum}\nolimits_i {{\int} {{\rm{d}}\overrightarrow r E_i^{ \bot \ast }} } \left( {\overrightarrow r \times \nabla } \right)A_i^ \bot + h.c. + \varepsilon _0{\int} {{\rm{d}}\overrightarrow r \overrightarrow E ^{ \bot \ast }} \times \overrightarrow A ^ \bot + h.c. \cr \,=\,\overrightarrow L + \overrightarrow S\end{array}$$

where $\overrightarrow E ^ \bot$ and $\overrightarrow A ^ \bot$ are the transverse components of the electric field and vector potential, respectively, and *i* denotes the order of the mode^15^. Note that any EM wave or field can be described as a superposition on the eigenbasis of the Laguerre–Gaussian modes: $\overrightarrow E ^ \bot = \mathop {\sum}\nolimits_{s,l} {P_{s,l}\overrightarrow E _{s,l}^ \bot }$ and $\overrightarrow A ^ \bot = \mathop {\sum}\nolimits_{s,l} {P_{s,l}\overrightarrow A _{s,l}^ \bot }$^16^, each of which carries the OAM of *lħ* depending on their azimuthal order *l* (*l* must be an integer) regardless of their spin state of ±*ħ* (*s* = ±1), where *P*_*s,l*_ represents the amplitude of each eigenmode. Due to mode orthogonality, the interference between different eigenmodes does not yield any additional OAM or spin, other than the intrinsic angular momentum associated with each mode. In this scenario, it is easily derived that the mean OAM and spin of an optical beam are (see “Materials and methods”):

3 $$\left\langle {L_Z} \right\rangle = \frac{{\mathop {\sum}\nolimits_{s,l} {\left| {P_{s,l}} \right|^2\hbar l} }}{{\mathop {\sum}\nolimits_{s,l} {\left| {P_{s,l}} \right|^2} }}\,{\mathrm{and}}\,\left\langle {S_Z} \right\rangle = \frac{{\mathop {\sum}\nolimits_{s,l} {\left| {P_{s,l}} \right|^2\hbar s} }}{{\mathop {\sum}\nolimits_{s,l} {\left| {P_{s,l}} \right|^2} }}$$

suggesting that the average OAM charge per photon in a complex field can take fractional numbers, i.e. FOAM. The ability of FOAM beams to carry any value between two (or multiple) integer numbers of quanta, as a result of superposition of two (or multiple) spatially variant fields of different vorticities, makes them especially useful in certain types of optical communications. For example, this feature leads to an increase in the modulation spectral efficiency, yielding higher bit rates for the same total bandwidth, as it can enable M-ary modulations in analogy to the well-established quadrature amplitude modulation scheme^17^.

The generation of optical beams carrying FOAM is similar to the simultaneous generation of at least two copropagating OAM beams of different amplitudes, mainly relying on carefully aligned tabletop optical set-ups involving a noninteger spiral phase plate, a geometric-phase-designed J plate, metasurfaces, or a spatial light modulator^18–24^. The desired FOAM can be generated by delicately tuning the weighting between two distinguished OAM components. For example, a reconfigurable FOAM transmitter has been recently demonstrated based on metagratings by varying illumination areas via a controllable aperture^25^. However, high-speed control and fast reconfigurability of FOAM, which is demanding in optical communication and computation applications, is challenging since the existing approaches are either static or mechanically slow despite several miniaturization efforts for on-chip OAM sources^26–28^. Ultrafast control based on nonresonant nonlinearities provides a viable route^29,30^; however, this approach requires relatively high control powers and shows relatively low switching efficiency. Here we overcome such limitations and demonstrate generation and fast all-optical control of FOAM light on the picosecond time scale by exploiting the transient carrier dynamics of the optical gain in a semiconductor vortex microlaser. Using a tuneable vortex microlaser platform we recently developed^31^ and a control laser pulse, spatiotemporal modulation of the spin–orbit interactions of counterpropagating longitudinal modes results in transient FOAM light generation. Our approach enables selective excitation and reconfiguration of the weighting of different vortex OAM components, leading to tuneable vector beams with a precise, continuous FOAM sweep between charge 0 and charge +2 within 100 ps.

## Results

**Fig. 1:**
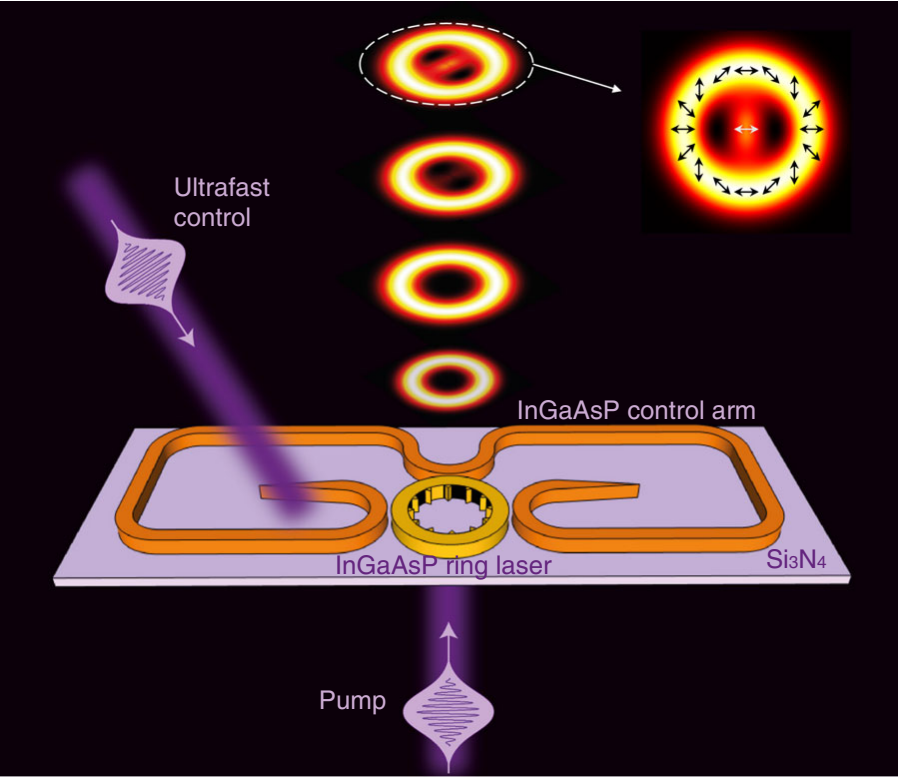
Ultrafast control of fractional orbital angular momentum (FOAM) by a tuneable vortex microlaser. An InGaAsP microlaser is embedded in a Si_3_N_4_ substrate and coupled with an external control arm, both pumped by ultrafast pulses, enabling the desired spin–orbit interactions. By controlling the time delay between the control and pump pulses, the FOAM of laser emissions can be temporally modulated with a picosecond resolution. The inset indicates the spatially varying electric field distribution showing the vectorial nature of the FOAM emission (see Supplementary Information for experimental measurements), assuming contributions from all four spin–OAM components in Eq. (4)

**Fig. 2:**
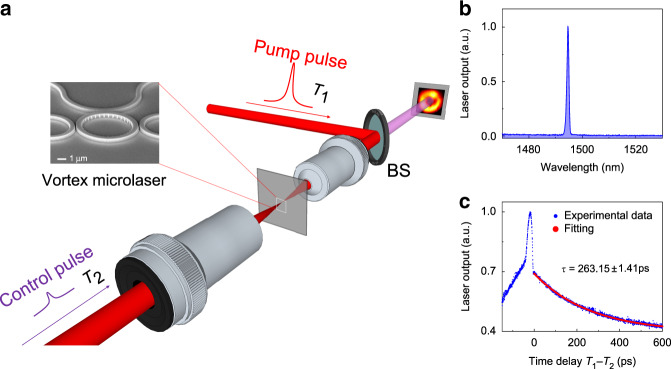
Experimental realization and characterization of ultrafast-controlled FOAM laser emissions. **a** Schematic of the experimental set-up, where two femtosecond pulses (i.e. pump and control) are projected onto the vortex microlaser using two microscope objectives (MOs), and the excited laser emission is imaged onto a CCD camera. The inset shows a scanning electron microscopic image of the tuneable vortex microlaser, where the angular grating is patterned at the inner sidewall to produce fractional OAM emission. **b** Spectrum of the FOAM emissions from the vortex microlaser, showing single-mode lasing at 1494.6 nm. **c** Measured and fitted temporal evolution of laser emissions, showing ultrafast gain dynamics in the applied InGaAsP multiple quantum wells with a carrier lifetime of 263.15 ± 1.41 ps (see Supplementary Information)

Integer OAM or FOAM beams are traditionally generated by the introduction of a phase variation into the transverse plane of an incident beam using carefully aligned optical elements, such as phase plates or metasurfaces. Alternatively, in integrated semiconductor nanophotonic circuits, the generation of vortex beams can rely on a different mechanism: robust selection of chiral resonant modes and their free space out-coupling with strategic phase matching conditions to convert the in-plane chiral modes into OAM vortex beams^26–28,31–33^. When the chiral modes are generated in active semiconductors (microlasers), owing to the relatively fast electron–carrier recombination dynamics of the gain medium, a series of high-speed modulation schemes can be applied to manoeuvre the in-plane chiral modes, which further facilitates ultrafast tunability and reconfigurability in OAM. To demonstrate FOAM control on the picosecond time scale, we consider our recently developed tuneable vortex microlaser^31^ consisting of a microring resonator and an external coupling loop with two control arms, all made of 200-nm-thick InGaAsP multiple quantum wells and embedded in a Si_3_N_4_ substrate (Fig. 1). The microring resonator supports two chiral modes: counterclockwise ($\circlearrowleft$) and clockwise ($\circlearrowright$) whispering gallery modes (WGMs) that are indirectly coupled by the external loop. The in-plane chiral modes are extracted and converted to OAM emissions by an angular grating with *M* equidistant scatters placed at the inner sidewall of the microring. In this work, we designed a microring resonator with a diameter of 7 μm and a width of 0.6 μm (see the inset in Fig. 2a). For the wavelength of approximately 1500 nm, the resonant order of the WGMs is *N* = 34, which, along with the diffraction order of the angular grating of *M* = 35, yields a total angular momentum of *J* = ∓1 for emissions extracted from the $\circlearrowleft$ and $\circlearrowright$ modes (see “Materials and methods”). The polarization state of extracted fields is, in general, a superposition of two transverse SAMs (i.e. left-handed (*s* = 1) and right-handed (*s* = −1) circular polarizations), with their amplitudes geometrically dependent on the dimensions of the microring. Therefore, the microlaser emissions contain 4 components corresponding to the 4 SAM–OAM combinations, described as:

4 $$\begin{array}{*{20}{l}}I_{{\rm{out}}} \,=\, I_{{\boldsymbol{L}}, - 2} + I_{{\boldsymbol{R}},0} + I_{{\boldsymbol{L}},0} + I_{{\boldsymbol{R}}, + 2} \hfill\\ \,\propto p_\circlearrowleft \sigma ^2 + p_\circlearrowleft \left( {1 - \sigma ^2} \right)+\, p_\circlearrowright \left( {1 - \sigma ^2} \right) + p_\circlearrowright \sigma ^2\hfill\end{array}$$

where *I*_out_ is the total intensity of the microlaser emissions, which is the sum of the intensities of 4 different spin–OAM components, with **L/R** indicating left/right-handed circular polarization and −2, 0, +2 denoting the associated OAM charge. The four spin–OAM components correspond to the out-couplings of the four combinations between the $\circlearrowleft$/$\circlearrowright$ mode and in-plane transverse spins, where $p_\circlearrowleft$/$p_\circlearrowright$ denotes the power associated with the $\circlearrowleft$/$\circlearrowright$ mode inside the cavity, respectively, and *σ* is the absolute value of the transverse SAM charge to describe the purity of the transverse spin. Equation (4) captures the central idea of this work—by exciting and reconfiguring the weighting of different OAM components, we demonstrate a precise, continuous FOAM sweep between charge 0 and charge +2 within 100 ps. The associated FOAM charge is calculated by integrating the OAM flux across the entire vector beam (see the spatially varying polarization state shown in the inset of Fig. 1)^34,35^, according to Eq. (3), given no spin–orbit coupling at the paraxial limit after the beam is emitted from the microlaser.

The WGM microlaser emission is controlled by exploiting indirect non-Hermitian mode coupling^36–39^ via suitable external loops^31^; see Fig. 1. The effective mode coupling can be turned from completely symmetric (Hermitian) to unidirectional (exceptional point), depending on the gain–loss contrast between the two control arms. This enables active tuning of the weighting between the two chiral modes (which is equivalent to the power ratio between $p_\circlearrowleft$ and $p_\circlearrowright$) (see “Materials and methods”). To achieve ultrafast control, we apply two synchronized femtosecond pulses from the same femtosecond laser (~140 fs) to pump the entire structure: a main pump pulse above the lasing threshold at time *T*_1_ to enable lasing oscillation in the microring resonator and a control pulse below the lasing threshold at time *T*_2_ to selectively excite gain carriers in one of the two arms. In addition to control of the peak gain via the pump power, here the transient carrier dynamics with a carrier lifetime typically of hundreds of picoseconds are the key to ultrafast control. As the excited carriers relax with time after pulse pumping, the associated gain coefficient also decays, as does the gain–loss contrast between the two control arms. Consequently, the non-Hermitian-controlled indirect coupling dynamically varies as a function of time, from unidirectional to symmetric. Hence, temporal overlap of the laser action in the microring and the time-varying optical gain in the control arm, precisely controlled by the time delay between the two femtosecond pump pulses, facilitates ultrafast control of the weighting between the two chiral modes in the microring and thus the weighting between different mode-converted OAM components in Eq. (4), yielding dynamic tuning of the FOAM of microlaser emissions with a picosecond resolution (Fig. 1).

In experiments, the emitted vortex beam was collected by a ×20 microscope objective and guided through a quarter waveplate and a linear polarizer for the desired polarization selection, and then its spatial profile was imaged onto a charge-coupled device (CCD) camera (Fig. 2a). The spectrum of the microlaser emissions confirms single-frequency laser action at the wavelength of 1494.6 nm with a sideband suppression ratio of >24 dB (Fig. 2b). To experimentally validate the transient carrier dynamics, we projected the control pulse to spatially overlap with the main pulse on the microring. The power of microlaser emissions, contributed by both pulses, was continuously captured as a function of the time delay between the two ultrafast pulses, i.e. *T*_1_ − *T*_2_ (Fig. 2c). If the two pulses are temporally far apart (i.e. $\left| {T_1 - T_2} \right| \gg 0$), then the emitted power is equivalent to the summation of the two individual laser actions/spontaneous emissions, as there is no temporal overlap of gain carriers excited by the two pulses. When the two pulses are close in time (not overlapped), the two pulses collectively boost the emitted power: for example, if the control pulse arrives first and a substantial amount of its excited carriers survive until the arrival of the main pulse, then the carriers excited by the two pulses augment each other, leading to significant enhancement in the power of the laser emissions. Nevertheless, the strongest enhancement does not occur at zero time delay (i.e. *T*_1_ − *T*_2_ = 0). In contrast, the first arrival of a strong pumping pulse depletes the carriers in the ground state, causing reduced absorption of the control pulse and thus leading to a sudden drop in the output power of the microlaser emissions^40,41^. The data afterwards exhibit a gain carrier lifetime of *τ* = 263.15 ± 1.41 ps in the InGaAsP multiple quantum wells, demonstrating the potential for ultrafast dynamic control (see Supplementary Information).

**Fig. 3:**
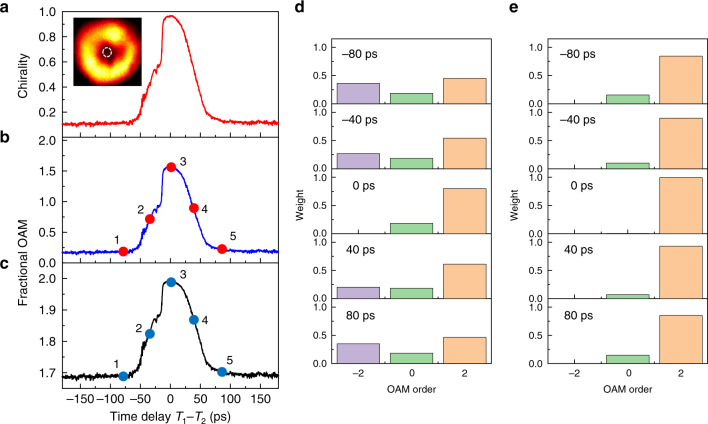
Temporal control of FOAM laser emissions. **a** Measured chirality of laser emissions as a function of the time delay between the pump and control pulses. The inset shows a snapshot of the vortex beam, where the dashed white circle area at the centre is used to analyse the chirality (see Supplementary Information). **b** Measured FOAM charge of laser emissions, which can be continuously tuned between 0.18 and 1.57 on a temporal scale of ~100 ps. **c** Measured FOAM charge of laser emissions after filtering out the cross-spin component, which can be continuously tuned between 1.68 and 2 on the same temporal scale. **d** OAM spectra of FOAM laser emissions at different time delays corresponding to the five points marked in **b**. The OAM charge −2/+2 component decreases/increases as the time delay approaches zero. **e** OAM spectra of FOAM laser emissions at different time delays corresponding to the five points marked in **c**. All cross-spin components (i.e. $\left| {L{\mathrm{,}} - 2} \right\rangle$ and $\left| {L{\mathrm{,}}0} \right\rangle$) are filtered out, and pure OAM charge 2 (i.e. $\left| {R, + 2} \right\rangle$) can be achieved at zero time delay

As discussed above, a precise, continuous FOAM sweep can be realized by exciting and reconfiguring the weighting of different OAM components. Ultrafast temporal control of the weighting between the two chiral modes and thus of the ratio between their associated powers ($p_\circlearrowleft$/$p_\circlearrowright$), which is the key dynamic parameter to reconfigure the fraction of each spin–OAM component in the emitted vector beam [see Eq. (4)], is achieved by tuning the temporal delay between the main pulse incident on the microring and the control pulse in the left control arm. In particular, the modulation of the carriers, facilitated by the precisely controlled temporal delay of the pulses in both the microring and the left control arm, enables ultrafast control of the fractional OAM charge. Note that *σ* is geometrically defined and fixed after the sample is fabricated. The power associated with all four spin–OAM components in Eq. (4) can be evaluated according to their spatial distributions and polarization states. For instance, the $\left| {R,0} \right\rangle$ and $\left| {L,0} \right\rangle$ states are located at the centre, as marked in the inset of Fig. 3a. By selectively capturing the power of each polarization state, the temporal response of lasing chirality, defined as $\left( {p_\circlearrowright - p_\circlearrowleft } \right)/\left( {p_\circlearrowright + p_\circlearrowleft } \right)$, is depicted in Fig. 3a (see Supplementary Information). Similarly, the temporally varying power associated with $\left| {R, + 2} \right\rangle$ and $\left| {L{\mathrm{,}} - 2} \right\rangle$ can be measured: as the time delay approaches zero, the chirality reaches 1 (i.e. $p_\circlearrowright \gg p_\circlearrowleft$), leading to the maximum weighting of $\left| {R, + 2} \right\rangle$ and zero $\left| {L{\mathrm{,}} - 2} \right\rangle$ (Fig. 3d, e). Altogether, by counting the power weighted average of all 4 integer OAM components, we conducted dynamic sweeping of the FOAM charge of the vector beam with a picosecond resolution, where the fractional charge could rapidly vary from 0.18 to 1.57 within 100 ps (Fig. 3b). The upper bound of the tuning range can be expanded if the cross-spin components are filtered. By selecting only the right-handed polarized components ($\left| {R{\mathrm{,}} + 2} \right\rangle$ and $\left| {R{\mathrm{,}}0} \right\rangle$), the FOAM charge varies in the range from 1.68 to 2 (Fig. 3c). Figure 3d, e show the histograms of decomposed integer OAM orders at five different time delays, with and without filtering the cross-spin components, respectively. Note that, because the $\left| {R{\mathrm{,}} + 2} \right\rangle$ and $\left| {L,0} \right\rangle$ states are both locked with the same $\circlearrowright$ mode, OAM charge 0 cannot vanish without filtering out the cross-spin components (Fig. 3d). The desired polarization selection further promotes the upper bound of the FOAM tuning to pure OAM charge +2, despite the generalized condition of a nonunitary transverse spin in our design (Fig. 3e). It is also worth noting that, due to the finite duration of the laser output pulse (<20 ps), the measured results correspond to the temporal average of the FOAM throughout the duration of the output pulse.

**Fig. 4:**
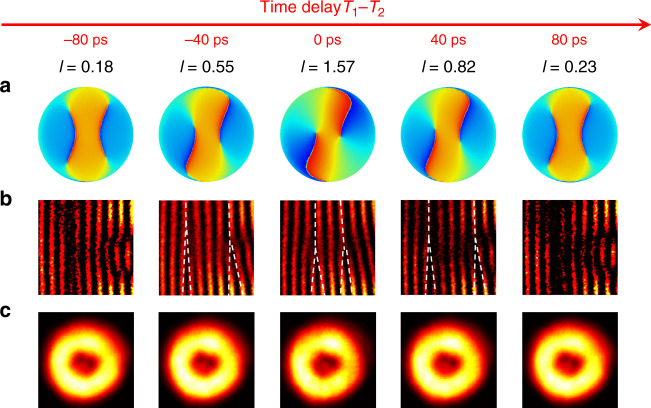
Temporal evolution of the FOAM laser emissions without filtering out the cross-spin components. **a** Simulated phase distribution *φ* of the elliptically polarized FOAMs at 5 different time delays between the pump and control pulses, carrying a fractional charge of *l* = 0.18, 0.55, 1.57, 0.82, and 0.23. Two singularity points associated with the FOAM vortex field emerge from the edges and move towards the centre as the delay time approaches zero. **b** Measured off-centre self-interferences of FOAM vortex fields at the five time delays. The images are cropped to show the fine details (see Supplementary Information). As the time delay approaches zero, forks arise from the two singularity points of the FOAM field and move towards the centre. Forks are marked with white dashed lines for better visibility. **c** Measured intensity maps of the FOAM field, featuring dark holes around a bright centre that arises from OAM charge 0 components (i.e. $\left| {R{\mathrm{,}}0} \right\rangle$ and $\left| {L{\mathrm{,}}0} \right\rangle$). The two dark holes become less visible when the delay time approaches zero, as they become spatially overlapped with only the $\left| {L{\mathrm{,}}0} \right\rangle$ component

**Fig. 5:**
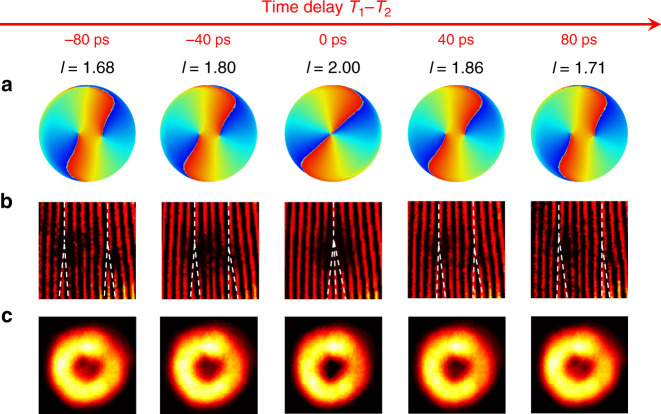
Temporal evolution of the FOAM laser emissions after filtering out the cross-spin components. **a** Simulated phase distribution *φ* of the right-handed circularly polarized FOAMs at 5 different time delays between the pump and control pulses, carrying a fractional charge of *l* = 1.68, 1.8, 2, 1.86, and 1.71. Two charge +1 singularity points associated with the FOAM vortex field move towards the centre and finally merge at zero time delay. **b** Measured off-centre self-interferences of FOAM vortex fields at the five time delays. The images are cropped to show the fine details (see Supplementary Information). Two separated charge +1 forks merge at zero time delay, where a single fringe splits into three, indicating an OAM charge of +2. Forks are marked with white dashed lines for better visibility. **c** Measured intensity maps of the FOAM field, where the bright centre arises from the same-spin OAM charge 0 component (i.e. $\left| {R{\mathrm{,}}0} \right\rangle$). As the two singularity points merge at the centre at zero time delay, the bright centre (i.e. $\left| {R{\mathrm{,}}0} \right\rangle$) vanishes, featuring a pure OAM charge of +2 (i.e. $\left| {R, + 2} \right\rangle$)

The phase singularity, associated with zero intensity at the singularity point, is a unique topological feature of the vortex beam. The ultrafast control enables novel space–time photonic transitions with dynamically evolving beam characteristics, as revealed by the splitting and merging of singularity points in the vector beam on the picosecond scale (Figs. 4 and 5). In the case without polarization selection (Fig. 4), all 4 spin–OAM components spatially superpose: $\left| {R{\mathrm{,}} + 2} \right\rangle$ and $\left| {L{\mathrm{,}} - 2} \right\rangle$ spatially overlap, but their opposite azimuthal phase variations cancel each other, while the two OAM charge 0 components ($\left| {R,0} \right\rangle$ and $\left| {L,0} \right\rangle$) carry planar phase fronts with a bright spot at the centre of the vortex beam at $\left| {T_1 - T_2} \right| \ge 80\,{\rm{ps}}$. Therefore, the microlaser emissions are nonchiral, and the simulated phase map does not indicate any pronounced phase singularity, consistent with the continuous interference fringes experimentally observed via the off-centre self-interference of the emitted vector beam. Note that the beam is in general elliptically polarized when including all four spin–OAM components. The phase of the beam in Fig. 4a is defined as $\varphi = {\mathrm{arg}}\left( {E^TS} \right)$, where *E* is the electric field vector and *S* is the unit elliptical polarization vector: $$S = \frac{1}{{\sqrt {\left| {E_x} \right|^2 + \left| {E_y} \right|^2} }}\left[ {\begin{array}{*{20}{c}} {\left| {E_x} \right|} \\ {\left| {E_y} \right|{\mathrm{e}}^{ - {\mathrm{i}}\alpha }} \end{array}} \right]$$, where *E*_*x*_ and *E*_*y*_ are the *x* and *y* components of the electric field and *α* is the phase delay between them. As $p_\circlearrowright$ prevails when the time delay between the two pulses approaches zero, $\left| {R{\mathrm{,}} + 2} \right\rangle$ and $\left| {L{\mathrm{,}}0} \right\rangle$ become dominant, leading to a dynamically increased FOAM charge. In a very short duration, two charge +1 singularity points emerge from the bright doughnut area and move towards the centre, as evidently shown in the measured interferograms: two forks are formed due to phase discontinuities at the two singularity points, where the fringe at each fork splits from one to two, indicating two charge 1 singularity points. The two charge +1 singularity points cannot touch because of the existence of $\left| {L{\mathrm{,}}0} \right\rangle$ arising from the geometrically defined *σ* associated with the nonunitary transverse spin. However, with successful filtering out of the cross-spin components ***L*** (Fig. 5), only $\left| {R{\mathrm{,}} + 2} \right\rangle$ survives at *T*_1_ − *T*_2_ = 0, so the two charge 1 singularity points merge to form a charge +2 singularity, manifested by a perfect doughnut with a 4*π* phase variation in the azimuthal direction. Since only the right-handed circularly polarized components remain, the phase of the beam in Fig. 5a is defined as $\varphi = {\mathrm{arg}}\left( {E^TR} \right)$, where *E* is the electric field vector and *R* is the unit vector of the right-handed circular polarization, $$R = \frac{1}{{\sqrt 2 }}\left[ {\begin{array}{*{20}{c}} 1 \\ {{\mathrm{e}}^{ - {\mathrm{i}}\pi /2}} \end{array}} \right]$$. In the off-centre self-interference of the emitted beam, the fork-like fringe splits from 1 to 3, confirming a topological charge of +2. When the two pump pulses do not temporally overlap (i.e. *T*_1_ − *T*_2_ ≠ 0), $\left| {R{\mathrm{,}} + 2} \right\rangle$ and $\left| {R{\mathrm{,}}0} \right\rangle$ coexist, also leading to a dynamically tuned FOAM charge with two charge +1 singularity points. In this case, the dynamic FOAM tuning ranges from 1.68 to 2, where the lower bound is mainly determined by the parameter *σ* given comparable $p_\circlearrowright$ and $p_\circlearrowleft$ at |*T*_1_ − *T*_2_ | ≥ 80 ps.

## Discussion

We have experimentally demonstrated ultrafast FOAM modulation in a continuously tuneable vortex microlaser, harnessing the fast transient carrier dynamics of the semiconductor optical gain in conjunction with non-Hermitian-controlled spin–orbital interactions of light. Notably, the switching speed of the FOAM vortex emission is in principle limited by the semiconductor optical response, with the potential to achieve sub-ps switching on other material platforms, such as perovskites^42^. The demonstrated FOAM tuning ranges from charge 0 to +2. However, our set-up can be easily modified to temporally coordinate ultrafast pumping on both control arms so that the vortex microlaser can in principle emit any FOAM state from charge −2 to +2, dynamically controlled with a picosecond resolution. Our scheme with ultrafast control of continuously tuneable FOAM, compatible with other modulation schemes in polarization, amplitude, frequency, etc., could be of potential relevance for the next generation of ultrahigh-speed optical communication systems, offering a feasible route to further enhancing the communication bandwidth based on multilevel OAM keying in coherent optical communication. Additionally, it could be exploited to reveal novel topological space–time features associated with pulsed vector beams^43,44^.

## Materials and methods

### Angular momentum associated with an EM wave

In the derivation, we apply the Coulomb gauge: $\nabla \cdot \overrightarrow A = 0$ and *ϕ* = 0 under the source-free condition. The vector potential of light radiated by our device can be expressed as^45^:

$$\begin{array}{*{20}{l}}\overrightarrow A ^ \bot \hfill&=\hfill& \mathop {\sum}\limits_{s,l} { - j^{l - 1}\frac{{PM}}{{R^3}}\frac{{k^2}}{{2\omega z}}} {\mathrm{{\Phi}}}\left( {\rho ,z} \right)J_{l - 1}\left( { - k\frac{\rho }{z}} \right)\exp\left[ {j\left( {l - 1} \right)\phi } \right]\overrightarrow e _s\hfill \\ \hfill&=\hfill& \mathop {\sum}\limits_{s,l} {P_{s,l}\overrightarrow A _{s,l}^ \bot },\hfill \end{array}$$

where *P* is the radiation intensity of each scatter, *M* is the number of scatters, *R* is the radius of the ring resonator, $$\begin{array}{l}P_{s,l} = \sqrt {\frac{{\varepsilon _0{\int} {\left| { - j^{l - 1}\frac{{PM}}{{R^3}}\frac{{k^2}}{{2z}}{\mathrm{{\Phi}}}\left( {\rho ,z} \right)J_{l - 1}\left( { - k\frac{\rho }{z}} \right)\exp \left[ {j\left( {l - 1} \right)\phi } \right]\overrightarrow e _s} \right|} ^2dV}}{{\hbar \omega }}} \end{array}$$ is the amplitude of each mode with normalization condition^46^ $\varepsilon _0{\int} {\left| {\overrightarrow A _{s,l}^ \bot } \right|} ^2dV = \frac{\hbar }{\omega }$, *k* is the wavevector, *ω* is the angular frequency, ${\mathrm{{\Phi}}}\left( {\rho ,z} \right) = {\mathrm{exp}}\left[ {jk\left( {z + \frac{{\rho ^2 + 1}}{{2z}}} \right)} \right]$ is a phase factor, *s* represents spin as aforementioned, and $\overrightarrow e _{ - 1} = (\overrightarrow e _x - i\overrightarrow e _y)/\sqrt 2$ and $\overrightarrow e _{ + 1} = \left( {\overrightarrow e _x + i\overrightarrow e _y} \right)/\sqrt 2$ are the unit vectors of right and left circular polarization, respectively. Correspondingly, the electric field can be expressed as^45^:

$$\begin{array}{*{20}{l}}\overrightarrow E ^ \bot \hfill&=\hfill& \mathop {\sum }\limits_{s,l} - j^l\frac{{k^2}}{{2z}}{\mathrm{{\Phi}}}\left( {\rho ,z} \right)J_{l - 1}\left( { - k\frac{\rho }{z}} \right)\exp \left[ {j\left( {l - 1} \right)\phi } \right]\overrightarrow e _s\hfill \\ \hfill&=\hfill& \mathop {\sum}\limits_{s,l} {P_{s,l}\overrightarrow E _{s,l}^ \bot }.\hfill \end{array}$$

To calculate the mean value of OAM and spin, note that:

$$\varepsilon _0{\int} {{{\rm{d}}\overrightarrow r E_{s1,l1,i}^ {\bot}}^ \ast \left( {\overrightarrow r \times \nabla } \right)A_{s2,l2,i}^ \bot } \propto {\int_0^{2\pi }} {e^{j\left( {l2 - l1} \right)\phi }{\rm{d}}\phi \overrightarrow e _{s1}^ \ast \cdot \overrightarrow e _{s2} = 2\pi \delta _{l1l2}\delta _{s1s2}},$$

$$\varepsilon _0{\int} {{{\rm{d}}\overrightarrow r \overrightarrow E _{s1,l1}^ \bot }}^ \ast \times \overrightarrow A _{s2,l2}^ \bot \propto {\int_0^{2\pi }} {e^{j\left( {l2 - l1} \right)\phi }{\rm{d}}\phi \overrightarrow e _{s1}^ \ast \times \overrightarrow e _{s2}} = 2\pi js_1\delta _{l1l2}\delta _{s1s2}\overrightarrow e _z.$$

With the OAM and spin associated with each individual mode:

$$\left( {\varepsilon _0\mathop {\sum}\limits_i {{\int} {{{\rm{d}}\overrightarrow r E_{s_1,l_1,i}^ \bot }}^ \ast } \left( {\overrightarrow r \times \nabla } \right)A_{s_1,l_1,i}^ \bot + h.c.} \right)_z = \hbar l_1,$$

$$\left( {\varepsilon _0{\int} {{{\rm{d}}\vec r\overrightarrow E _{s_1,l_1}^ \bot }}^ \ast \times \overrightarrow A _{s_1,l_1}^ \bot + h.c.} \right)_z = \hbar s_1,$$

we find the mean OAM and mean spin of the field as described in Eq. (3).

### Ultrafast non-Hermitian chiral control

In the case of pumping the main microring and the left control arm as implemented in our experiment (Fig. 1), the ultrafast chiral response of our tuneable vortex microlaser can be described by the following coupled mode equations when *T*_1_ − *T*_2_ ≥ 0 by assuming a fast response of carriers:

$$\frac{{{\rm{d}}E_\circlearrowright }}{{{\rm{d}}t}} = i\omega E_\circlearrowright + ( {\kappa _0 + \kappa e^{ - \gamma }e^{\gamma _Le ^{- ({T1 - T2})/{\tau }}}} )E_\circlearrowleft,$$

$$\frac{{{\rm{d}}E_\circlearrowleft }}{{{\rm{d}}t}} = i\omega E_\circlearrowleft + \left( {\kappa _0 + \kappa e^{ - \gamma }} \right)E_\circlearrowright,$$

where $E_\circlearrowright$/$E_\circlearrowleft$ denotes the electric field amplitude of the $\circlearrowright$/$\circlearrowleft$ mode, respectively; *κ* is the coupling between $\circlearrowright$ and $\circlearrowleft$ modes without gain or loss; *κ*_0_ corresponds to the coupling arising from the nonlinear effects and fabrication imperfections; −*γ* is the single pass attenuation through the control waveguide; *γ*_L_ indicates the single pass amplification through the control waveguide at the time of pulse incidence; and *τ* is the lifetime of gain carriers in the control waveguide. The power ratio between the two chiral modes circulating in the microring can be roughly estimated from the steady-state linear supermode analysis and reads as:

$$\frac{{P_\circlearrowright }}{{P_\circlearrowleft }} = \left( {\frac{{E_\circlearrowright }}{{E_\circlearrowleft }}} \right)^2 = \frac{{\kappa _0 + \kappa e^{ - \gamma }e^{\gamma _Le ^{- \left( {T_1 - T_2} \right)/\tau }}}}{{\kappa _0 + \kappa e^{ - \gamma }}}.$$

Note that the carrier dynamics are assumed to have an instantaneous and linear impact on the chiral ratio because the pump pulse duration is several orders of magnitude shorter than the carrier lifetime, while the lasing pulse duration is shorter than *τ*. Numerical analysis of laser pulse dynamics, based on semiconductor rate equations^36^ with spontaneous emission noise, shows that the ratio of pulse energies between the two chiral modes circulating in the ring is well described by the above equation.

### Design of transverse spin

The transverse spin, in which the electric field rotates around the axis perpendicular to the propagation direction of light^47,48^, is designed by inspecting the value of $\frac{{\left\langle {E{\mathrm{|}}R} \right\rangle }}{{\left| E \right|}}$ through full-wave numerical simulations, where *E* denotes the electric field vector and *R* denotes the unit vector of right-hand circular polarization. With the geometric configuration of the microring, 200 nm in height and 600 nm in width, $\frac{{\left\langle {E{\mathrm{|}}R} \right\rangle }}{{\left| E \right|}}$ is 0.9, which is equivalent to *σ* = 0.9, indicating the purity of the transverse spin.

### Extraction of OAM

An angular grating of order *M* is placed at the inner sidewall of the microring to extract the *N*th order WGMs. The local phase at the *q*th scatter position can be written as $\varphi _{q,{\rm{local}}} = 2\pi Cq\left( {N - M} \right)/M$, where $q \in \left\{ {0,\,M - 1} \right\}$ and *C* = ±1 for the $\circlearrowleft$ and $\circlearrowright$ modes. Due to the nonunitary transverse spin, the extracted global phases experience opposite coordinate rotation and can be expressed as $\varphi _{{\mathrm{global}},q} = 2\pi C\left[ {q\left( {N - M} \right) - q} \right]/M$ for the left-handed spin (*s* = 1) component and $\varphi _{{\mathrm{global}},q} = 2\pi C\left[ {q\left( {N - M} \right) - q} \right]/M$ for the right-handed spin (*s* = −1) component. Therefore, the linearly varying phase distribution creates an emission with two integer OAM modes with topological charges of $C\left( {N - M - 1} \right)$ and $C\left( {N - M + 1} \right)$ with orthogonal spin states and a total angular momentum |*J*| = |*N* − *M*|.

### Sample fabrication and characterization

The tuneable vortex microlaser was fabricated using electron-beam lithography, inductively coupled plasma etching (ICP), plasma-enhanced chemical vapour deposition (PECVD), and substrate transfer techniques. Hydrogen silsesquioxane (HSQ) in methyl isobutyl ketone (MIBK) solution was used for patterning. The concentration ratio of HSQ (FOX15) to MIBK was adjusted such that an adequately thick layer of resist was formed for etching after exposure and development. The patterned wafer was then immersed and slightly stirred in tetramethylammonium hydroxide solution (MFCD-26) and rinsed in deionized water. The chip was subsequently processed by ICP dry etching with BCl_3_ and Ar, followed by removal of HSQ resist using buffered oxide etchant. Next, a Si_3_N_4_ cladding was deposited by PECVD. The wafer was then bonded to a glass slide, which functioned as a base. Finally, the InP substrate was removed by wet etching with a mixture of HCl and H_3_PO_4_.

The fabricated tuneable vortex microlaser was pumped on the front side by a femtosecond pulsed laser with an 80 MHz repetition rate and a 140 fs pulse duration at a wavelength of 800 nm. The pump power was controlled with a variable neutral density filter, monitored by a power meter. The pump pulse was reflected by a 50:50 beam splitter and focussed onto the microring cavity using a Mitutoyo ×20 near-infrared long-working-distance objective (NA = 0.4). The additional control pump pulse was tuned with a half waveplate together with a polarization beam splitter and projected onto the control arms through a Mitutoyo ×10 long-working-distance objective (NA = 0.28) from the backside (through the SiN substrate). The delay time between these two pulses was controlled by a motorized linear translation stage. The laser emission from the front side was collected by the aforementioned ×20 microscope objective, guided through a quarter waveplate and a linear polarizer for the desired polarization selection, and then recorded on a CCD camera. A Michelson interferometer was built to measure the off-centre self-interference of the beam to analyse the beam characteristics.

## Supplementary information

Supplementary Information
