# Supplementary material for: Ultrafast control of fractional orbital angular momentum of microlaser emissions
Source: Light Sci Appl. 2020 Oct 21;9:179. doi: 10.1038/s41377-020-00415-3 (PMC7576132; doi:10.1038/s41377-020-00415-3)
Supplement: Supplementary file 1 — Supplementary Information [file 41377_2020_415_MOESM1_ESM.pdf]

Supplementary Information

Ultrafast control of fractional orbital angular momentum of microlaser emissions

Zhifeng Zhang[1], Haoqi Zhao[1], Danilo Gomes Pires[2], Xingdu Qiao[1], Zihe Gao[3], Josep M. Jornet[4], Stefano Longhi[5,6], Natalia M. Litchinitser[2], and Liang Feng[3,1]*

[1]*Department of Electrical and Systems Engineering, University of Pennsylvania, Philadelphia, PA 19104, USA*

[2]*Department of Electrical and Computer Engineering, Duke University, Durham, NC 27708, USA*

[3]*Department of Materials Science and Engineering, University of Pennsylvania, Philadelphia, PA 19104, USA*

[4]*Department of Electrical and Computer Engineering, Northeastern University, Boston, MA 02115, USA*

[5]*Dipartimento di Fisica, Politecnico di Milano and Istituto di Fotonica e Nanotecnologie del Consiglio Nazionale delle Ricerche, Piazza L. da Vinci 32, Milano I-20133, Italy*

[6]*Instituto de Fisica Interdisciplinar y Sistemas Complejos IFISC (CSIC-UIB) - Palma de Mallorca, Spain*

*Email: fenglia@seas.upenn.edu

1. Polarization state of microlaser emissions

When only the microring laser is pumped, the microlaser emissions contain all 4 spin-OAM components as described in Eq. (4) and form a vector beam whose polarization state spatially varies as shown in the inset of Fig. 1 and marked in Fig. S1a. The observed bright circle corresponds to the contributions from the OAM ±2 components and the non-zero center arises from the two OAM 0 components. The spatially varying polarization nature of the beam was verified by selecting two orthogonal linear polarizations, showing two complementary 4-lobe patterns (Fig. S1b and Fig. S1c). Since the field at the center of the beam is horizontally polarized, it is visible when a linear polarizer is horizontally placed but varnishes when the polarizer is vertically aligned.

a b c

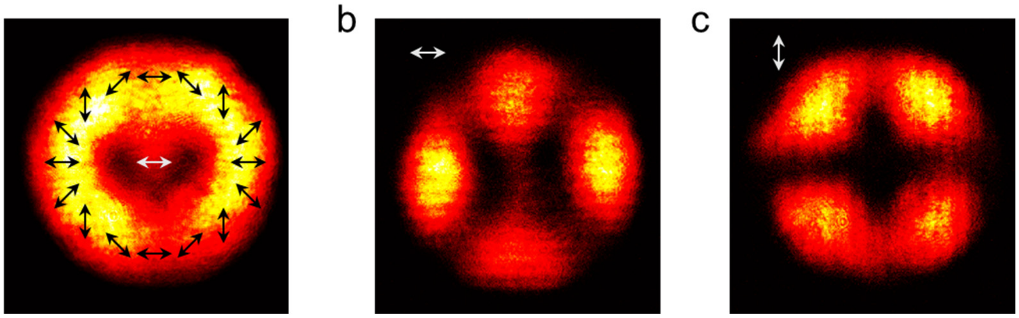

Figure S1. a. Measured intensity pattern of the microlaser emissions with arrows indicating the polarization states. **b.** Measured intensity pattern of the microlaser emissions passing through a linear polarizer in the horizontal direction, showing 4 main lobes with a central line connecting the upper and lower lobes. **c.** Measured intensity pattern of the microlaser emissions passing through a linear polarizer in the vertical direction, showing only 4 lobes 90° rotated with respect to those in **b**.

2. Measurement of carrier lifetime

The measurement of carrier lifetime, as shown in Fig. 2c, was carried out by focusing both the main pump and control pulses onto the microring resonator and collecting the lasing emissions as a function of time delays between the two pulses. The energy of the main pump pulse is much greater than the lasing threshold, while the energy of the control pulse is lower than the lasing threshold. The polarization states of the two pulses are orthogonal, so they do not interfere and serve as two individual pumps. Note that at each time delay, the measurement corresponds to temporal integration of microlaser emissions. We also conducted two control experiments in additional to that shown in Fig. 2c: 1) both pulses' energies are much greater than the lasing threshold and 2) both pulses' energies are less

than the lasing threshold but their summation is greater than the threshold when overlapped in time and space. (Fig. S2)

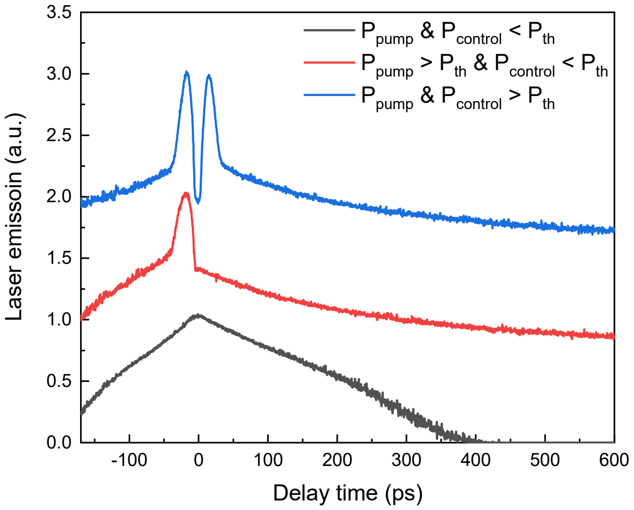

Figure S2. Measured lasing emission intensities when the microring is pumped by different combinations of energies associated with the main pump pulse and the control pulse.

When both pulses' energies are lower than the lasing threshold, lasing only occurs within a certain range of $T_1 - T_2$. If $T_1 - T_2 > 0$, the control pulse reaches the laser cavity first and excites a number of carriers decaying in time, but some carriers remain upon the arrival of the pump pulse. In this scenario, if the sum of the carriers contributed by both pulses surpasses the lasing threshold, the laser action happens until the total carriers decay to drop below the lasing threshold.

When pump pulse has a greater energy above the lasing threshold but the control pulse is below the threshold, the laser action is always measured regardless of the time delay between the two pulses. In the region of $T_1 - T_2 < 0$, however, there are three small sub-regions exhibiting different features: (i) < -38 ps (the left kink), (ii) between -38 and -17 ps (peak) and (iii) between -17 and 0 ps. In region (i), the lasing triggered by the main pump pulse ends completely and its excited carriers drop below the threshold. However, the arrival of the control pulse brings the microring back to lasing since a good number of carriers remain. As a result, the captured signal, including a main lasing pulse excited by the main pump and a secondary lasing pulse triggered by the control, is enhanced but with a lower slope efficiency. In region (ii), the lasing emission is enhanced more rapidly and efficiently. Despite the decay of the carriers, the lasing action triggered by the main pump

pulse is still on-going. In this scenario, the control pulse directly contributes to the laser actions with a greater slope efficiency. The peak signal corresponds to the right time delay where the remaining carriers excited by the main pump, together with the new carriers supplied by the control, reach the maximum absorption of pulse energy corresponding to the power saturation threshold, below which the microlaser has the best slope efficiency. In this case, the carriers excited by the control pulse is converted into laser emissions with the highest efficiency, resulting in the maximum enhancement in laser emissions in time. In region (iii), a very large number of carriers are excited by the main pulse and still remain till the arrival of the control pulse. In this case, the carriers on the ground state are depleted, causing reduced absorption of the control pulse and therefore bringing the microlaser into the power saturation region with reduced energy conversion efficiency. This leads to a drop in microlaser emissions with a minimum occurring at the time when the two pulses are completely overlapped. In the region of $T_1 - T_2 > 0$, the control pulse arrives earlier and excites carriers with a lifetime τ (no lasing occurs yet). Since the control pulse is below the lasing threshold, only the spontaneous emission occurs before the arrival of the main pump pulse, where the carriers decay at the spontaneous recombination rate. The remaining carriers effectively enhance the total laser output. When $T_1 - T_2$ increases, this effective enhancement decreases in its intensity with the spontaneous recombination rate, by which data fitting was performed to retrieve the carrier lifetime: τ ~ 263 ps.

When both pulses are greater than the lasing threshold with similar energy, we observed a symmetric response of output laser emissions with respect to zero time delay. Similarly, the three small regions also exist, as the aforementioned lasing mechanism still applies in this case. Note that, in the region of $T_1 - T_2$ is sufficiently large, the carriers excited by the control pulse decay to a level similar to the weak control pulse discussed above. In this case, data fitting was also performed to retrieve the carrier lifetime, which shows a similar result: τ ~ 249 ps.

3. Measurement and calculation of lasing chirality

The chirality of the microlaser can be evaluated by $Chirality = \frac{p_{\circlearrowright} - p_{\circlearrowleft}}{p_{\circlearrowright} + p_{\circlearrowleft}}$. To calculate the chirality, we performed two integrations of the power in the region defined by the white dashed circle in the inset of Fig. 3a (i.e., the OAM 0 components), with (i) filtering out the

left-handed circularly polarized components and (ii) filtering out the right-handed circularly polarized components, respectively. The measured powers from these two different conditions are proportional to $p_{\circlearrowleft}$ and $p_{\circlearrowright}$, respectively, given the same out-coupling efficiency (see Eq. (4) in the main text).

4. <u>Measurement and crop of off-center self-interferences patterns</u>

The off-center self-interference patterns were performed to validate the charge of the microlaser emissions, as shown in Fig. S3, showing 2 pairs of forks: one pair both facing up in the white box on the left half and the other pair both facing down on the right half. The opposite directions of the two pairs show their origins from the two beams in the interference. To make the results more visible and manifest detailed information, we only cropped the part in the white box and showed in Fig. 4 and Fig. 5.

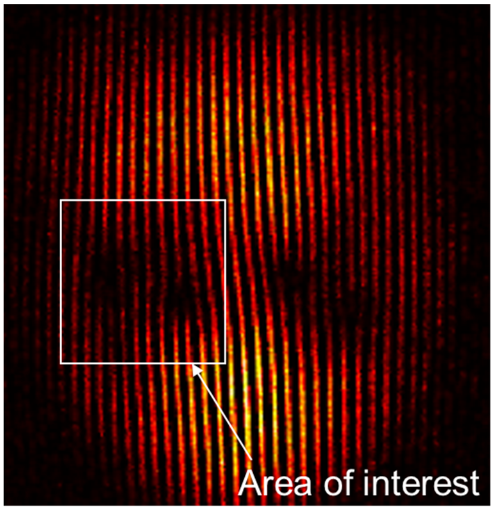

Figure S3. Full image of one off-center self-interference pattern. The white box region was cropped and zoomed in to show the details in the main text. In this full image, four single charge forks (2 pairs) are observed with a pair on each side and the 2 pairs face in opposite directions.
